# Reference gene selection for normalization of RT-qPCR gene expression data from *Actinidia deliciosa* leaves infected with *Pseudomonas syringae* pv. *actinidiae*

**DOI:** 10.1038/srep16961

**Published:** 2015-11-19

**Authors:** Milena Petriccione, Francesco Mastrobuoni, Luigi Zampella, Marco Scortichini

**Affiliations:** 1 Consiglio per la ricerca in agricoltura e l’analisi dell’economia agraria (CREA), Unità di ricerca per la Frutticoltura, Via Torrino 3, I-81100 Caserta, Italy

## Abstract

Normalization of data, by choosing the appropriate reference genes (RGs), is fundamental for obtaining reliable results in reverse transcription-quantitative PCR (RT-qPCR). In this study, we assessed *Actinidia deliciosa* leaves inoculated with two doses of *Pseudomonas syringae* pv. *actinidiae* during a period of 13 days for the expression profile of nine candidate RGs. Their expression stability was calculated using four algorithms: geNorm, NormFinder, BestKeeper and the deltaCt method. Glyceraldehyde-3-phosphate dehydrogenase (*GAPDH*) and protein phosphatase 2A (*PP2A*) were the most stable genes, while β-tubulin and 7s-globulin were the less stable. Expression analysis of three target genes, chosen for RGs validation, encoding the reactive oxygen species scavenging enzymes ascorbate peroxidase (APX), superoxide dismutase (SOD) and catalase (CAT) indicated that a combination of stable RGs, such as *GAPDH* and *PP2A*, can lead to an accurate quantification of the expression levels of such target genes. The *APX* level varied during the experiment time course and according to the inoculum doses, whereas both *SOD* and *CAT* resulted down-regulated during the first four days, and up-regulated afterwards, irrespective of inoculum dose. These results can be useful for better elucidating the molecular interaction in the *A. deliciosa*/*P. s*. pv. *actinidiae* pathosystem and for RGs selection in bacteria-plant pathosystems.

Gene expression analysis is an important tool that is employed to elucidate the complex regulatory networks of the genetic, signalling and metabolic pathway mechanisms that underlie plant-pathogen interactions^1^. Although gene expression microarrays are an ideal tool to provide a snapshot of the global gene transcriptional activity, reverse transcription quantitative real-time polymerase chain reaction (RT-qPCR) assays are normally used to verify results on a smaller scale due to its high sensitivity, high specificity and good reproducibility^2^. RT-qPCR is used to determine the fold change of the expression of genes of interest (GOIs) or as a technique to confirm the results of differential protein studies obtained by proteomic analyses^3^. However, to improve RT-qPCR accuracy and reliability, a strategy aimed at normalizing gene expression data is required. Normalization can correct the variation associated with variability occurring in the experimental procedures (i.e., sample quantification errors, variation between samples)^4^,^5^. Generally, the use of reference genes (RGs) as internal controls is the most common method for normalizing data in gene expression experiments, so each RGs should be validated for particular tissue, cell and experimental conditions. RGs are internal controls that are exposed to assay conditions similar to the gene of interest. Ideally, the expression profile of the RGs should not be influenced by the conditions of the experiment. Usually, the main candidate RGs are involved in general cell metabolism pathways and are widely used in plant, animal and fungal systems^6^,^7^,^8^,^9^,^10^. In some plant pathosystems, transcriptomic technologies, such as microarray, qPCR and RT-qPCR, have identified the molecular mechanisms that are activated upon infection and maintained during pathogenesis that are responsible for disease symptom development, resistance and basal defence^1^,^11^,^12^. Moreover, several studies have analysed the stability of candidate RGs in some pathosystems, demonstrating that some RGs show a certain degree of variability. In fact, plant pathogens, such as viruses, bacteria and fungi, can induce metabolic alterations and gene expression reprogramming in different organs of the host plant, thus modifying the expression of RGs^13^,^14^,^15^,^16^. There are few studies aimed at identifying putative RGs that can be used in transcriptomic analyses for plant pathogenic bacteria, including *Citrus* spp., such as *Xanthomonas citri*^17^, *Xylella fastidiosa* and Candidatus *Liberibacter asiaticus*^18^, and potato, such as *Pectobacterium atrosepticum*^19^. Regarding phytopathogenic pseudomonads, a study has been performed with the model plant *Arabidopsis thaliana* and virulent and avirulent strains of *Pseudomonas syringae*^6^. *P. s.* pv. *actinidiae* (PSA) is the causal agent for bacterial canker of both the yellow-fleshed (*Actinidia chinensis*) and green-fleshed (*A. deliciosa*) kiwifruit, and it is currently causing severe economic losses worldwide^20^. This pathogen, most probably, originated in China^21^,^22^,^23^ and shows a diverse population structure^24^. Proteomic studies revealed the involvement of multiple classes of proteins that are differentially expressed by the plant and the pathogen occurring over a period of weeks after inoculation, as demonstrated by its necrotic or hemibiotrophic phase^25^,^26^. So far, detailed transcriptomic analyses performed during the early stages of plant colonization have not be released, and a selection of RGs for the normalization of qPCR and RT-qPCR gene expression would be useful to standardize and compare the data. In this study, several algorithms such as geNorm^27^, NormFinder^28^, BestKeeper^29^ and the deltaCt method^30^ were employed to assess the expression stability of nine RGs belong to different functional and abundance classes to significantly reduce the chance that they are co-regulated. Four of these genes were used in previous studies as RGs for kiwifruit: actin (*ACT*)^31^,^32^, eukaryotic elongation factor 1α (*eEF-1a*)^33^, protein phosphatase 2A (*PP2A*)^33^ and ubiquitin conjugating enzyme 9 (*UBC9*)^34^. Other genes were described as RGs, either to pathosystems or biotic stress: glyceraldehyde-3-phosphate dehydrogenase (*GAPDH*)^35^, SAND family protein (*SAND*)^13^,^16^,^36^, β-tubulin (*TUB*)^14^,^36^, 7s-globulin (*GLO7A*)^37^ and cyclophilin (*CYP*)^38^,^39^.

Here, we assessed nine candidate RGs for RT-qPCR normalization of gene expression in *A. deliciosa* leaves during the first period of interaction (13 dpi) with PSA.

## Results

### Multiplication and growth of *P. s.* pv. *actinidiae* in *A. deliciosa* leaf

During the time course of the experiment, the multiplication and growth of PSA CRA-FRU 8.43 inoculated at 1-2 × 10^3^cfu/ml and 1-2 × 10^*7*^cfu/ml into *A. deliciosa* cv. Hayward leaves was assessed. When inoculated at the lower dose, the growth of the pathogen within the leaf never exceeded 10^5^cfu/ml and showed a peak of 2-3 × 10^4^cfu/ml at nine dpi; no symptoms were observed in the inoculated leaves. By contrast, when the pathogen was inoculated at 1-2 × 10^7^cfu/ml, it incited the appearance of tiny necrotic spots on many of the inoculated leaves of nine dpi (see Supplementary Fig. S1 online). In this case, only the green tissue was precisely removed to prepare the samples.

### Selection of candidate reference genes, amplification specificity and efficiency

Nine RGs, commonly used as internal controls for expression studies in other pathosystem, were screened in *A. deliciosa* leaves inoculated with the pandemic PSA strain CRA-FRU 8.43. To determine the specificity of the primer pairs used in this study, melting curve analysis and agarose gel electrophoresis were performed following the RT-qPCR experiment. A single peak in the obtained melting curve confirmed the specificity of the amplicon, and no signal was detected in the negative controls for all of the tested RGs (see Supplementary Fig. S2 online). In addition, a single band with the expected size was detected in a single PCR product (see Supplementary Fig. S3 online). The standard curve method using a pool of all of the cDNA samples was performed to calculate the PCR efficiency (E) and the correlation coefficient (R2) of each primer pair. Average E values ranged from 100.7 to 108.2%, with R^2^ varying from 0.991 to 0.999 (Table 1). The results showed that all of the primer pairs were suitable for RT-qPCR analysis.

### Expression levels of the reference genes

**Figure 1:**
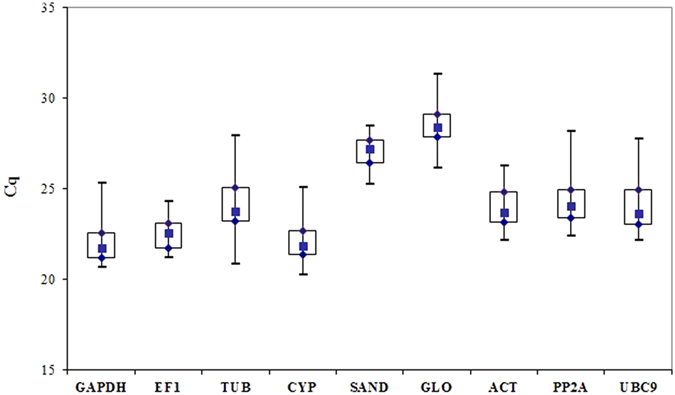
Cq values for nine candidate reference genes across experimental samples. A square across the box is depicted as the median. The box indicates the 25th and 75th percentiles and the whiskers caps represent the maximum and minimum values.

RT-qPCR was used to quantify the mRNA levels of nine candidate RGs, and the expression stability was investigated. To determine the expression levels of the candidate RGs, the raw quantification cycle (Cq) values were determined. The nine candidate RGs displayed a wide expression range, with Cq ranging from 20.23 to 31.31, across all of the tested samples, with mean Cq values between 22.06 ± 0.92 and 28.58 ± 0.86 (Fig. 1). All of the tested RGs showed a normal distribution in Cq values according to the Kolmogorov and Smirnov method. These genes were clearly distributed into different expression level categories. The results showed that *CYP* was the most expressed gene with the lowest mean Cq (22.06). On the other hand, *GLO7A* was the least expressed gene with the highest mean Cq value (28.58). *TUB* showed the most variation in expression level among the evaluated RGs by the larger whisker taps and boxes compared to the other genes, suggesting its low stability. Most of the candidate RGs were highly expressed, with average Cq values between 22 and 24 cycles, except *SAND* and *GLO7A*, which showed average Cq values at intermediate expression levels (Fig. 1).

### Expression stability of the reference genes

Four different statistical applets (geNorm, NormFinder, BestKeeper and the deltaCt method) were used to evaluate the stability of expression of selected RGs. The analyses were performed for three comparison groups considering both low- and high-dose bacterial inocula in the leaves and their combined dataset. In each comparison group, the nine RGs were ranked from the most stable to the least stable. The data obtained from biological replicates were analysed separately to verify that the variation was not due to the treatment, but was intrinsic to the gene itself^40^,^41^.

### Genorm Analysis

**Figure 2:**
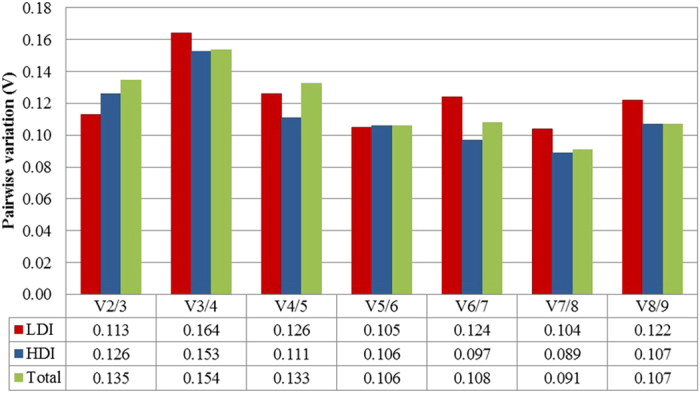
Pairwise variation (V_n_/V_n + 1_) analysis between the normalization factors (NF_n_ and NF_n + 1_) was performed by the geNorm program to determine the optimal number of reference genes required for effective normalization, and carried out for qPCR data normalization in leaves inoculated with low dose of *Pseudomonas syringae* pv. *actinidiae* inoculum (LDI), leaves inoculated with high dose of *P. s.* pv. *actinidiae* inoculum (HDI) and all samples combined together (Total).

Nine RGs were ranked in three comparison groups based on their average expression stability (M-value), as shown in Tables 2, 3 and 4. All of the tested RGs showed an overall limited variance, with M-values lower than 1.5, which was the default limit (M≤1.5), indicating a high stability level of the analysed genes in our experimental conditions. *GAPDH*, *PP2A* and *UBC* were the three most stable genes in this pathosystem, with slight differences in ranking for three comparison groups. In A. deliciosa leaves inoculated with a low dose of bacterial inoculum, *GAPDH* was the most stable gene (Table 2), while in leaves inoculated with a high dose of bacterial inoculum and when all of the sample sets were analysed together, *PP2A* was the most stable gene (Tables 3 and 4). *TUB* was the least stable gene in three comparison groups (Tables 2, 3 and 4). In this study we used the geNorm algorithm to find the optimal number of suitable RGs required for proper normalization. In three comparison groups, geNorm analysis revealed that by step wise calculation the pairwise variation value V2/3 was lower than the threshold value (0.15), suggesting that two RGs could be used for normalization under these conditions (Fig. 2). This suggested that the optimal number of RGs for normalization was two and that the addition of the third RGs showed no significant effect on the normalization of gene expression. Finally, *GAPDH* and *PP2A* were identified as the best RGs and selected for normalization by geNorm.

### NormFinder analysis

NormFinder ranks the RGs according to their stability values under the tested conditions. The results of NormFinder analysis were slightly different from those of geNorm. However, in the three comparison groups, *GAPDH* emerged as the most stably expressed gene with the lowest stability value. *GAPDH* and *PP2A* still occupied the next two top positions for higher stability when we considered the total dataset (Table 4) or in *A. deliciosa* leaves inoculated with a high dose of bacterial inoculum (Table 3), while in *A. deliciosa* leaves inoculated with a low dose of bacterial inoculum, *GAPDH* and *ACT* were the most stable RGs (Table 2). The NormFinder results indicated that *TUB* was the least stable RG in the total dataset, confirming our geNorm results.

### BestKeeper analysis

The results of BestKeeper analysis were reported in Tables 2, 3 and 4. In the total dataset, BestKeeper analysis highlighted six RGs characterized by the least overall variation, with SD < 1; *SAND* and *eEF-1a* were the most stable genes, with SD values of 0.69 and 0.76, respectively (p < 0.001) (Table 4). In *A. deliciosa* leaves with a low dose of bacterial inoculum, *SAND* (0.72) was the most stable gene, followed by *eEF-1a* and *GLO7A*, with SD values of 0.81 and 0.95, respectively (Table 2). In kiwifruit leaves with a high dose of bacterial inoculum, BestKeeper revealed that only the expression of *TUB* overcame the stability threshold; *CYP* and *GAPDH* were considered to be the most stable genes, with SD values of 0.50 and 0.61, respectively (Table 3).

**deltaCt method** 

The results of the deltaCt method were reported in Tables 2, 3 and 4. *GAPDH* was the most stable gene for the three comparison groups. For the entire dataset, the results were similar to NormFinder and geNorm analysis, with *GAPDH* and *PP2A* as the top two ranked RGs, with a slight difference in the ranking (Table 4). *TUB* was the least stable gene in three comparison groups, as demonstrated by other statistical algorithms.

In this study, to determine the consistency of the ranks of candidate RGs produced by geNorm, NormFinder, BestKeeper and the deltaCt method, the Pearson correlation coefficient was employed (Table 5). The Pearson correlations achieved from the calculations were positive and significant for all methods, except BestKeeper. The most significant correlation of the rank of all RGs ranked by two methods was geNorm and deltaCt in *A. deliciosa* leaves inoculated with a high dose of bacterial inoculum (r = 0.958), followed by NormFinder *vs.* deltaCt in *A. deliciosa* leaves inoculated with a low dose of bacterial inoculum (r = 0.895) (Table 5).

For the overall final ranking obtained by the four algorithms, the two top RGs for the total dataset were *GAPDH* and *PP2A*, while the least stable were *GLO7A* and *TUB*.

### Expression analysis of the target genes for reference gene validation

The expression of three genes encoding the reactive oxygen species (ROS) scavenging enzymes ascorbate peroxidase (APX), superoxide dismutase (SOD) and catalase (CAT), induced during the systemic infection of kiwifruit leaves with PSA, were chosen to further validate the reliability of the selected RGs for the normalization of RT-qPCR data. In this study, we followed two normalization strategies to determine the expression of these target genes. The first used the best two RGs (*GAPDH* and *PP2A*) given by ranking from four methods (geNorm, BestKeeper, NormFinder and deltaCt), and the second used the least stable RGs (*TUB* and *GLO7A*).

**Figure 3:**
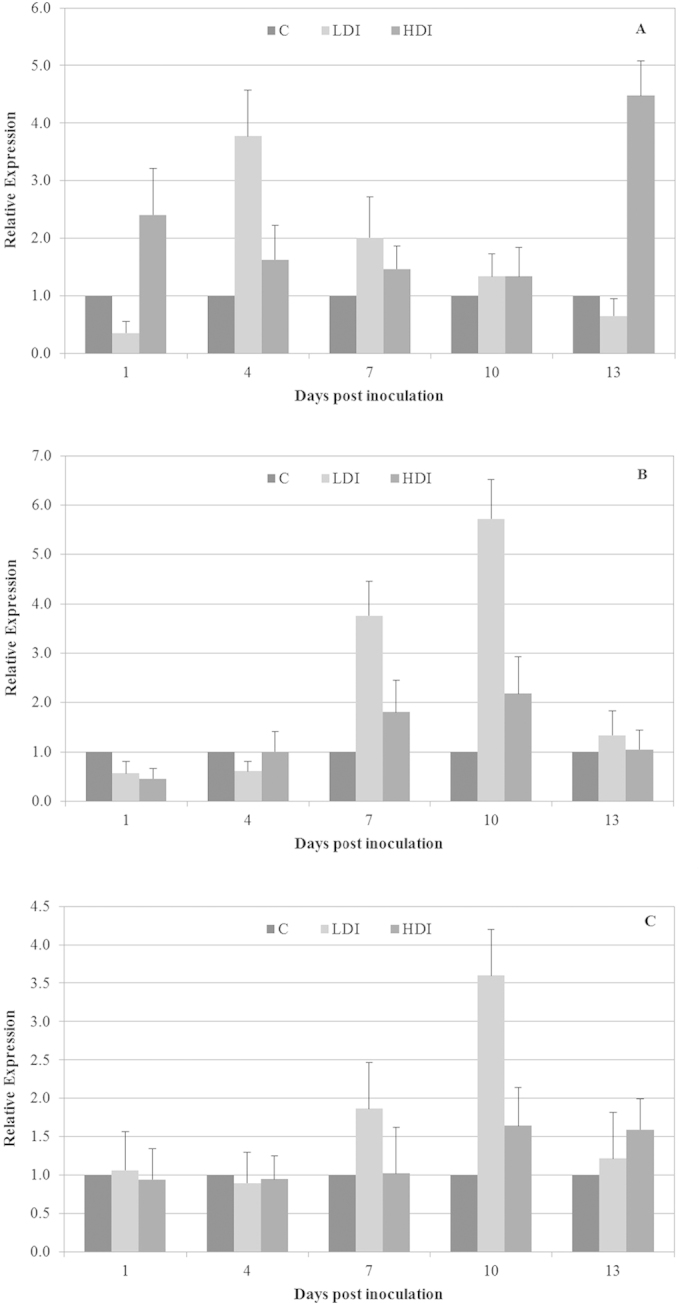
Relative expression of the *ascorbate peroxidase* (A), *catalase* (B), and *superoxide dismutase* (C) gene normalized with the most stable reference genes (*glyceraldehyde-3-phosphate dehydrogenase* and *protein phosphatase 2*) in kiwifruit leaves inoculated with low dose of *Pseudomonas syringae* pv. *actinidiae* inoculum (LDI), leaves inoculated with high dose of *P. s.* pv. *actinidiae* inoculum (HDI) compared to the mock-inoculated plants (C). Data are expressed as mean ± standard deviation.

In *A. deliciosa* leaves inoculated with a high dose of bacterial inoculum, an up-regulation in *APX* mRNA expression was observed during the time course of the experiment with 2.4- and 4.5-fold changes after 1 and 13 dpi, respectively. Instead, when we used a low dose of bacterial inoculum, we observed an accumulation of the *APX* transcript after 4 dpi with a 3.7-fold-change and a gradual decrease from 7 to 13 dpi (Fig. 3A)

**Figure 4:**
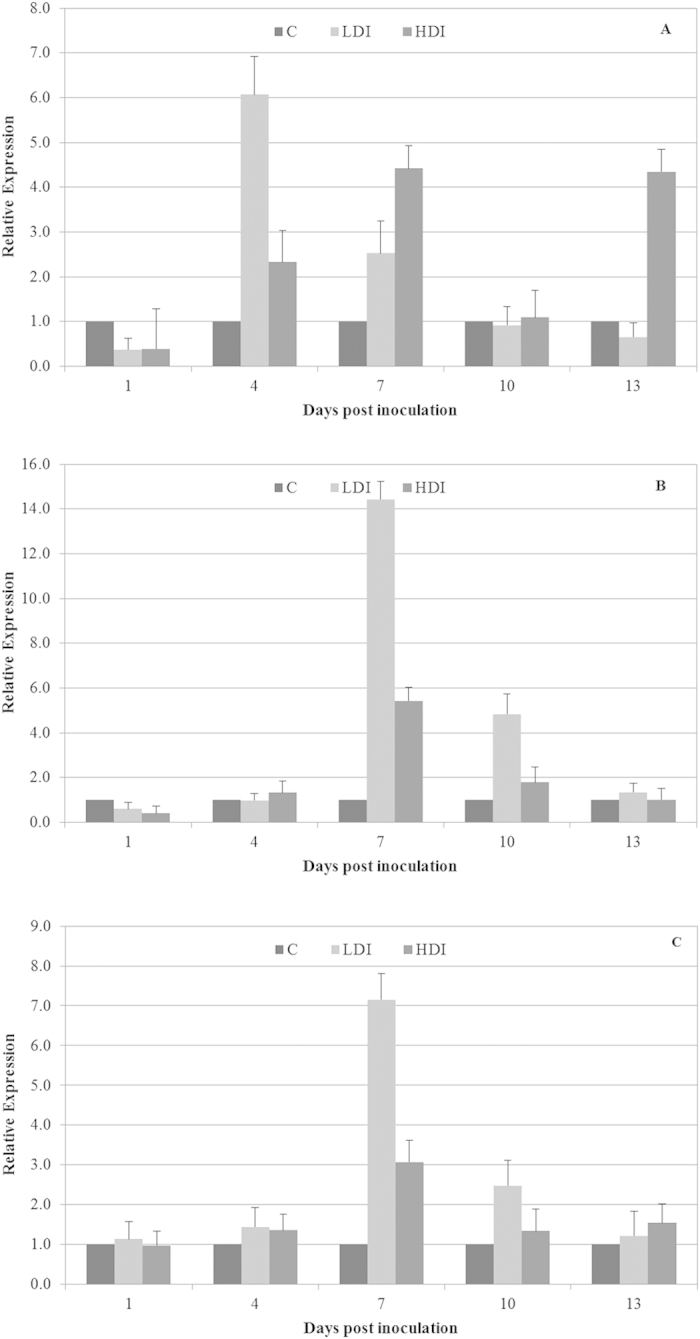
Relative expression of the *ascorbate peroxidase* (A), *catalase* (B), and *superoxide dismutase* (C) gene normalized with the least stable reference genes (*tubulin* and *7s-globulin*) in kiwifruit leaves inoculated with low dose of *Pseudomonas syringae* pv. *actinidiae* inoculum (LDI), leaves inoculated with high dose of *P. s.* pv. *actinidiae* inoculum (HDI) compared to the mock-inoculated plants (C). Data are expressed as mean ± standard deviation.

A down-regulation in *CAT* mRNA expression during the first 4 dpi was observed, and subsequently, we registered a gradual up-regulation in *CAT* mRNA expression in *A. deliciosa* leaves inoculated with a low- and high-dose of bacterial inocula. The maximum level of the transcript was reached after 10 dpi, with a 2.2- and 5.7-fold change in infected leaves with high- and low-dose bacterial inocula, respectively (Fig. 3B). Similarly, we observed in the accumulation of the *SOD* transcript, that the maximum average value after 10 dpi was a 3.6- and 1.6-fold change, with low and high bacterial inocula, respectively (Fig. 3C). Our results confirm that the transcriptional levels of *APX*, *CAT* and *SOD* are subjected to complex regulation in PSA-infected kiwifruit leaves. This information is distorted when we normalize against the least stable genes, upon which the expression levels of *APX*, *CAT* and *SOD* were inaccurate and altered transcriptional profiles were displayed (Fig. 4).

## Discussion

In research of plant molecular pathology, studies on gene expression patterns are important for understanding the biological process involved in host-plant interactions. Presently, several methods can be applied to study gene expression levels, but RT-qPCR has become the primary quantitative method for the high-throughput and accurate expressing profiling of target genes. For RT-qPCR analysis, the requirement of a normalization method against RGs is important to achieve reliable results. As suggesting by the “Minimum Information for publication of Quantitative real-time PCR Experiments” (MIQE) guidelines^4^, the use of RGs as internal controls is the most appropriate normalization strategy^7^. Ideal RGs should be stably expressed in all cells or tissues and remain stable under different experimental conditions^42^. Several studies highlighted that there is neither a universal RG nor a defined number of genes to use, but the choice and an optimal number of RGs should be experimentally determined^4^,^27^. Many reliable RGs have been determined in plant cells and across different plant species, developmental stages, and biotic and abiotic stresses^43^. However, to the best of our knowledge, few studies have been carried out to assess RGs in bacteria-plant pathosystems^6^. Here, we assessed nine RGs for their use as internal controls in gene expression studies of the *A. deliciosa* response to infection by PSA upon leaf infiltration using two different doses of bacterial inoculum. To identify the best RGs, four different statistical algorithms were used. Combined use of geNorm, NormFinder, BestKeeper and the deltaCt method to select and validate the best RGs generated substantial discrepancies in the final ranking due to different mathematical models associated with each algorithm, as confirmed by other studies^18^,^44^,^45^. As reported in other studies, the most discrepant results in gene stability ranking were obtained with BestKeeper^46^.

In the total dataset, *PP2A*, *GAPDH* and *UBC* were identified as the top three RGs using geNorm, while *GAPDH*, *PP2A* and *ACT* were suggested as the most stable RGs by NormFinder and the deltaCt method. According to BestKeeper, *ACT*, *GAPDH*, *PP2A* and *UBC* were ranked fifth to eighth, respectively. Among all of the tested RGs, *TUB* was ranked as the least stable gene in the four statistical algorithms, and its use as a RG should be avoided in RT-qPCR experiments in this pathosystem. To overcome differences in the ranking of RGs, we adopted the geometric mean of all four algorithms to obtain a final ranking^47^.

As suggested by several studies, the accuracy of RT-qPCR can improve by using more than one RG^27^. The optimal number of candidate RGs for normalization of RT-qPCR data has been evaluated by geNorm software. Our results showed a pairwise variation V2/3 value below 0.15, which indicates that combination of two-RGs was sufficient for optimal normalization in the three comparison groups.

The final ranking showed that the two top RGs for the total dataset were *GAPDH* and *PP2A* and can be used as RGs for RT-qPCR normalization in this pathosystem. *GAPDH* was indicated to be a stable RG in a tomato-virus interaction^15^, in virus-infected mammalian cells^48^ and in wheat infected with barley yellow dwarf virus (BYDV)^14^, but was the least stable RG in *Coffea* spp. hypocotyls inoculated with *Colletrichum kahawae*^13^. *PP2A* was a stable RG in virus-infected leaf tissues of *Nicotiana benthiamiana*^48^ and in virus-infected *Arabidopsis thaliana*^43^. In our study, *UBC* was among the four most stable RGs, as demonstrated in *Coffea arabica* leaves inoculated with *Hemileia vastarix*^49^, but was considered to be the least stable RG in common bean inoculated with *Colletotrichum lindemuthianum*^50^. *TUB* was not confirmed as a stable normalization factor in our conditions, confirming our previous proteomic study that showed the variability of this protein in *A. chinensis* shoot during systemic infection with PSA^25^; however, in other pathosystems, such as *Puccinia graminis* f sp. *tritici*-infected wheat, *TUB* was one of the most stable RGs^51^. Furthermore, this RG showed highly variable expression levels in closely related cereals, such as wheat, barley and oat infected with BYDV; *TUB* was unstable in wheat and reasonably stable in two other species^14^. The *SAND* transcript was ranked lower among RGs in our pathosystem than was identified in *Nicotiana benthiamiana* and *Lycopersicum esculentum* plants inoculated with viruses^52^,^53^. These variations in the expression profiles of RGs in different pathosystems confirm the need for validation for RGs under each specific condition. Some RGs can be involved in different metabolic pathways^13^ and influenced in a plant tissue-dependent manner during plant-pathogen interactions^15^.

The suitability of the selected RGs has been evaluated analysing the expression levels in three target genes (*APX*, *CAT* and *SOD*) that encode for proteins that are directly involved in ROS detoxification, protecting cells from oxidative bursts induced as responses to pathogen invasion^54^. *SOD* catalyses the dismutation of O_2_^–^ to H_2_O_2_, *CAT* dismutates H_2_O_2_ to oxygen and water, and *APX* reduces H_2_O_2_ to water by utilizing ascorbate as a specific electron donor^55^. The balance between *SOD* and *APX* or *CAT* activities in cells is crucial for determining the steady-state level of O_2_^–^ and H_2_O_2_. In our study, the accumulation of *APX*, *CAT* and *SOD* gene transcripts was strongly influenced by the dose of bacterial inoculum used. Indeed, these genes involved in ROS detoxification and the oxidative-stress response have a key role for bacteria survival and pathogenesis^55^. The *APX* up-regulation during a relatively long time course of infection (i.e., 10 days) was observed upon the twig inoculation with the same high dose of PSA CRA-FRU 8.43 also in the case of *A. chinensis* “Soreli”^25^. In the same study, however, neither *CAT* nor *SOD* were found differentially expressed 10 days after the twig inoculation. Irrespective of the inoculum doses, both *CAT* and *SOD* resulted up-regulated during the first four days of infection, and, subsequently, their level in the leaf tissues declined. Interestingly, a similar trend was observed for *SOD* in the *Phaseolus vulgaris*/*P. s*. pv. *phaseolicola* pathosystem after the inoculation of bean leaves with the same high dose of bacterial inoculum used in the present study (i.e., 1 × 10 ^7^ cfu/ml)^56^. In this study, however, the *SOD* level in the bean primary leaves and into the apoplastic fluid starts to decrease 48 and 24 hours after the artificial inoculation.

Furthermore, in this study, we demonstrated that to correctly quantify *APX*, *CAT* and *SOD*, it was necessary to choose the RGs that had transcript levels that were not influenced by bacterial infections and that the use of inappropriate RGs can markedly change the expression pattern of a given target gene, leading to incorrect results.

This is the first study in which a set of candidate RGs was analysed in terms of their expression stability in *A. deliciosa* leaves infected with PSA. Four different statistical algorithms showed slight differences in the final ranking of RGs, but by combining and analysing the data together, we demonstrated that two genes, *GAPDH* and *PP2A*, are the most stably expressed transcripts in all infected kiwifruit leaves.

The validation of RGs in our study provides new information that will be useful for a better understanding of the molecular mechanisms implicated in the expression profiles of target genes in the *A. deliciosa*/*P.s.* pv *actinidiae* pathosystem. It should be considered that ideal RGs can vary with the pathosystem under investigation, and therefore, these genes should be carefully selected for each study conforming to the MIQE guidelines.

## Methods

### Plant material, *P. syringae* pv. *actinidiae* inoculations and experimental design

Two-year-old, self-rooted, pot-cultivated *A. deliciosa* “Hayward” plants and the pandemic PSA strain CRA-FRU 8.43 were used in this study^21^. This bacterial strain was originally isolated from *A. chinensis* leaf spot and further characterized^57^,^58^. Plants were maintained in an aseptic room with 95% relative humidity with natural light and no further fertilization after their transfer from the nursery. They were watered regularly. Inoculation took place in spring (i.e., May). The strain was grown for 48 h on nutrient agar (Oxoid) with 3% sucrose added (NSA) at 25 ± 1 °C. Subsequently, a low (1-2 × 10^3^cfu/ml) and high (1-2 × 10^7^cfu/ml) dose of bacterial inoculum, determined using spectrophotometry, were prepared in sterile, distilled water. To avoid wounding, the inoculation occurred by gently spraying the suspensions on the abaxial surface of fully expanded, healthy, young leaves, until the appearance of homogenous water-soaked areas on the whole leaf lamina. Twenty plants per dose were inoculated. Artificial inoculations were performed separately, according to the dose. Control plants were treated in the same way with sterile, distilled water. After inoculation, plants were maintained separately and were kept for 24 h in a moist chamber (100% humidity), which was required for optimal infection. During the experiment, the multiplication and growth of the pathogen was assessed as previously described^21^. Leaves were collected after one day post-inoculation and at intervals of three days for 13 days, immediately frozen in liquid nitrogen and stored at −80 °C until RNA isolation. In the same treatment group (inoculated and mock inoculated), each biological replicate was obtained by pooling three leaves from different plants harvested at random. Three independent biological replicates were performed for each sample with three technical replicates each.

### Total RNA extraction and cDNA synthesis

Total RNA was isolated from *A. deliciosa* leaves inoculated with PSA as well as from control leaves as described by Rubio-Piña and Zapata-Perez^59^. Residual genomic DNA was digested by RNase-free DNase (Invitrogen Life Technologies, Carlsbad, CA, USA) according to the manufacturer’s instructions. The RNA concentration was quantified by measuring the absorbance at 260 nm using a Jasco V-530 UV/VIS spectrophotometer (Tokyo, Japan). The purity of all of the RNA samples was assessed at an absorbance ratio of OD260/280 and OD260/230, while its structural integrity was checked by agarose gel electrophoresis. Only high-quality RNA with OD 260/280 and OD 260/230 > 2 was used for subsequent steps. Single-stranded cDNA was synthesized from 1 μg of total RNA using an iScript™ Select cDNA Synthesis Kit and oligo(dT)20 primers (Bio-Rad, Milan, Italy), according to the manufacturer’s instructions.

### Selection of candidate reference genes, PCR primer design and amplification efficiency test

For this study, special attention was paid to a select set of nine candidate RGs (*ACT, eEF-1a, PP2A, UBC9, SAND, TUB, GLO7A, CYP* and *GAPDH*) to investigate their robustness as internal controls for RT-qPCR in *A. deliciosa*. These genes belong to different functional and abundance classes to significantly reduce the chance that they are co-regulated. *APX*, *CAT* and *SOD* were selected as genes of interest. Gene-specific primers, such as *SAND*, *TUB*, *GLO7A*, *CYP*, *GAPDH*, *APX*, *CAT* and *SOD*, were designed in our laboratory using Primer Expression software version 3 (Table 1). The amplification efficiency of each candidate/target gene was determined using a pool representing all of the cDNA samples. First, all of the primers were examined by end-point PCR, all of the chosen candidates/target were expressed, and specific amplification was confirmed by a single band of appropriate size in a 2% agarose gel after electrophoresis (see Supplementary Fig. S3 online). In a second step, the pool was used to generate a five-point standard curve based on a ten-fold dilution series. The amplification efficiency (E) and correlation coefficient (R^2^) of the primers were calculated from the slope of the standard curve according to the equation^60^:


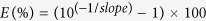


### Quantitative Real-time PCR (qPCR)

Quantitative Real-time-PCR was performed using a CFX Connect Real-time PCR Detection System (Bio-Rad) to analyse the specific expression of each reference/target gene. cDNA was amplified in 96-well plates using the SsoAdvanced™ SYBR® Green Supermix (Bio-Rad), 15 ng of cDNA and 300 nM specific sense and anti-sense primers in a final volume of 20 μl for each well. Thermal cycling was performed, starting with an initial step at 95 °C for 180 s, followed by 40 cycles of denaturation at 95 °C for 10 s and primer-dependent annealing (Table 1) for 30 s. Each run was completed with a melting curve analysis to confirm the specificity of amplification and lack of primer dimers.

### Determination of reference gene expression stability

Data analyses were performed on three groups: a) infected plants with a low dose of bacterial inoculum compared to the mock-inoculated plants dataset (LDI), b) infected plants with a high dose of bacterial inoculum compared to the mock-inoculated plants dataset (HDI), and c) the entire dataset (Total). The stability of candidate RGs for several comparison groups was analysed with the following four applets: geNorm^27^, NormFinder^28^, BestKeeper^29^ and the deltaCt method^30^. The raw Cq values were converted into relative quantities and imported into the geNorm and NormFinder software programs; no transformed Cq values are required for BestKeeper and the deltaCt method. GeNorm calculates an expression stability value (M) for each RG and then determines the pairwise variation (V) of each RG with all of the other genes. At the end of analysis, by stepwise exclusion of the gene with the highest M-value (less stable), this tool allows for the ranking of the tested RGs according to their expression stability. The optimal number of RGs required for normalization was determined by pairwise variation V_n_/V_n + 1_ (0.15 recommended threshold).

NormFinder calculates the expression stability value (SV) for each gene, taking into account intra- and inter-group variations of the samples set^28^. A low SV-value indicates the high expression stability of this gene.

BestKeeper is an Excel-based software tool that selects best-suited RGs by performing a statistical analysis based on Pearson correlation coefficient (*r*), standard deviation (SD) a coefficient of variance (CV). Only genes with a high *r* value and a low SD are combined into BestKeeper index (BKI) value using the geometric mean of their Cq values. Finally, this tool determines the correlation coefficient of each candidate RG with the BKI value, along with the probability (*p*) value. The RG with the highest coefficient of correlation with the BKI is considered to be the most stable. The deltaCt (dCt) method compares relative expression of pairs of RGs within each sample to identify stable RGs^30^. A ranking of the RGs using the four algorithms together was obtained as suggested by Velada *et al.*^47^. Correlations among the stability values of RGs obtained with different software were analysed using Pearson’s correlations (P < 0.05 and P < 0.01). All statistical analyses were performed using the SPSS v. 20.0.

### Validation of reference genes

To confirm the reliability of the RGs, the relative expression profiles of *APX*, *CAT* and *SOD* genes were determined and normalized with the most stable and less stable genes. Relative fold changes in gene expression was calculated using the comparative 2^−ΔΔCt^ method and normalized to the corresponding RGs levels^29^,^61^.

### Statistical analysis

Data are displays as mean ± standard deviation. Cq values were tested for normality (Kolmogorov-Smirnov test) prior to analysis. Statistical analysis of data was performed by one-way ANOVA followed by LSD *post-hoc* test. Calculation were performed using the SPSS v. 20.0.

## Additional Information

**How to cite this article**: Petriccione, M. *et al.* Reference gene selection for normalization of RT-qPCR gene expression data from *Actinidia deliciosa *leaves infected with *Pseudomonas syringae *pv. *actinidiae*. *Sci. Rep.* **5**, 16961; doi: 10.1038/srep16961 (2015).

## Supplementary Material

Supplementary Information

## Figures and Tables

**Table 1:** Descriptions of nine candidate reference genes in *Actinidia deliciosa* and parameters derived from RT-qPCR analysis.

Gene name (Gene symbol) AccessionNumber	Primer sequence (5′-3′)	Amplicon length (bp)	Ta (°C)	Tm (°C)	PCR efficiency (%)	Regression Coefficient (R^2^)	Reference
**Reference genes**							
**Actin (*****ACT***)	F: CCAAGGCCAACAGAGAGAAG	198	59	83	108.2	0.991	Ledger et al. 2010
**FG440519**	R: GACGGAGGATAGCATGAGGA						
**Ubiquitin conjugating enzyme 9 (*****UBC9***)	F: CCATTTCCAAGGTGTTGCTT	109	59	84	100.7	0.999	Gunther et al. 2011
**FG409482**	R: TACTTGTTCCGGTCCGTCTT						
**Elongation factor 1α (*****eEF-1a***)	F: GCACTGTCATTGATGCTCCT	118	59	82.5	102.3	0.999	Nardozza et al., 2013
**FG418280**	R: CCAGCTTCAAAACCACCAGT						
**Protein phosphatase 2A (*****PP2A***)	F: GCAGCACATAATTCCACAGG	110	59	80.5	102.3	0.999	Nardozza et al.2013
**FG522516**	R: TTTCTGAGCCCATAACAGGAG						
**SAND family protein (*****SAND***)	F: TGCGTCTGAGATTGAGGAGG	90	59	82	105.8	0.995	This study
**FG475049**	R: GCCGTTTGAGAATCCGACAT						
**Tubulin (*****TUB***)	F: CCGTTGCATCTTGGTACTGC	92	59	81	104.2	0.997	This study
**FG411346**	R: GGGAGAAGGAATGGACGAGA						
**7s-globulin (*****GLO7A***)	F: CCCCAGCTACCAGAAAGTGA	101	59	85.5	102.2	0.998	This study
**FG438711**	R: GATTCTGGTCGTTGGAAGCG						
**Cyclophilin (*****CYP***)	F: ATCTGCGAGAATGCCAAACC	84	59	85.5	106.7	0.998	This study
**FG412294**	R: TACAGCTCCATCACGATCCG						
**Glyceraldehyde-3-phosphate dehydrogenase (*****GAPDH***)	F: GTTCCCACTGTCGATGTCTCA	112	59	82	102.5	0.999	This study
**EU281570**	R: CCCTTCATCTTGCCCTCAGA						
**Target genes**							
**Ascorbate peroxidase (*****APX***)	F: GGAGCCGATCAAGGAACAGT	102	59	81.5	101.2	0.997	This study
**FG408540**	R: AACGGAATATCAGGGCCTCC						
**Catalase (*****CAT***)	F: GCTTGGACCCAACTATCTGC	108	59	82.5	100.9	0.999	This study
**FG470670**	R: TTGACCTCCTCATCCCTGTG						
**Superoxide dismutase (*****SOD***)	F: CACAAGAAGCACCACCAGAC	106	59	86	103.5	0.998	This study
**FG471220**	R: TCTGCAATTTGACGACGGTG						

**Table 2:** Average stability values (SV) of the nine candidate reference genes are shown for leaves inoculated with low dose of *Pseudomonas syringae* pv. *actinidiae* inoculum.

Rank	geNorm		NormFinder		BestKeeper		deltaCt	
	Gene	SV	Gene	SV	Gene	SV	Gene	SV
1	*GAPDH*	0.38	*GAPDH*	0.09	*SAND*	0.72	*GAPDH*	0.64
2	*PP2A*	0.38	*ACT*	0.18	*eEF-1a*	0.81	*UBC9*	0.72
3	*UBC9*	0.38	*UBC9*	0.19	*GLO7A*	0.95	*ACT*	0.74
4	*CYP*	0.53	*PP2A*	0.23	*CYP*	0.97	*PP2A*	0.76
5	*ACT*	0.61	*GLO7A*	0.26	*ACT*	1.02	*eEF-1a*	0.77
6	*eEF-1a*	0.64	*eEF-1a*	0.31	*GAPDH*	1.16	*CYP*	0.83
7	*SAND*	0.72	*CYP*	0.31	*UBC9*	1.28	*GLO7A*	0.97
8	*GLO7A*	0.78	*SAND*	0.40	*PP2A*	1.34	*SAND*	1.02
9	*TUB*	0.86	*TUB*	0.49	*TUB*	1.68	*TUB*	1.12

**Table 3:** Average stability values (SV) of the nine candidate reference genes are shown for leaves inoculated with high dose of *Pseudomonas syringae* pv. *actinidiae* inoculum.

Rank	geNorm		NormFinder		BestKeeper		deltaCt	
	Gene	SV	Gene	SV	Gene	SV	Gene	SV
1	*PP2A*	0.40	*GAPDH*	0.09	*CYP*	0.50	*GAPDH*	0.63
2	*GAPDH*	0.41	*PP2A*	0.12	*GAPDH*	0.61	*PP2A*	0.64
3	*UBC9*	0.42	*eEF-1a*	0.13	*eEF-1a*	0.63	*UBC9*	0.64
4	*eEF-1a*	0.53	*UBC9*	0.14	*SAND*	0.65	*eEF-1a*	0.66
5	*ACT*	0.57	*ACT*	0.17	*UBC9*	0.68	*ACT*	0.73
6	*CYP*	0.63	*SAND*	0.21	*GLO7A*	0.68	*CYP*	0.82
7	*SAND*	0.67	*TUB*	0.27	*PP2A*	0.76	*GLO7A*	0.85
8	*GLO7A*	0.71	*GLO7A*	0.30	*ACT*	0.83	*SAND*	0.85
9	*TUB*	0.78	*CYP*	0.39	*TUB*	1.14	*TUB*	1.00

**Table 4:** Average stability values (SV) of the nine candidate reference genes are shown for leaves inoculated with low and high dose of *Pseudomonas syringae* pv. *actinidiae* inoculum.

Rank	geNorm		NormFinder		BestKeeper		deltaCt	
	Gene	SV	Gene	SV	Gene	SV	Gene	SV
1	*PP2A*	0.39	*GAPDH*	0.09	*SAND*	0.69	*GAPDH*	0.65
2	*GAPDH*	0.41	*PP2A*	0.17	*eEF-1a*	0.76	*PP2A*	0.69
3	*UBC9*	0.42	*ACT*	0.20	*GLO7A*	0.86	*ACT*	0.73
4	*CYP*	0.54	*UBC9*	0.22	*CYP*	0.92	*UBC9*	0.73
5	*ACT*	0.63	*GLO7A*	0.28	*ACT*	0.95	*eEF-1a*	0.75
6	*eEF-1a*	0.66	*eEF-1a*	0.29	*GAPDH*	0.97	*CYP*	0.82
7	*SAND*	0.71	*CYP*	0.33	*PP2A*	1.08	*GLO7A*	0.89
8	*GLO7A*	0.75	*SAND*	0.33	*UBC9*	1.17	*SAND*	0.92
9	*TUB*	0.81	*TUB*	0.40	*TUB*	1.42	*TUB*	1.02

**Table 5:** Correlation of the candidate reference genes rank according to the evaluation based on four statistical algorithms for three comparison groups (leaves inoculated with low dose of *Pseudomonas syringae* pv. *actinidiae* inoculum (LDI), leaves inoculated with high dose of *P. s.* pv. *actinidiae* inoculum (HDI) and all samples combined together (Total)).

Rank	Correlation		
	LDI	HDI	Total
geNorm VS NormFinder	0,764*	0,745*	0,782*
geNorm VS BestKeeper	−0.066	0,448	−0,070
geNorm VS deltaCt	0,877**	0,958**	0,859**
NormFinder VS BestKeeper	0,122	0,017	0.099
NormFinder VS deltaCt	0,895**	0,749*	0.894*
deltaCt VS BestKeeper	0,135	0,542	0,187

^*^Correlation is significant at 95% confidence level (2-tailed).

^**^Correlation is significant at 99% confidence level (2-tailed).
