# Supplementary material for: Reference gene selection for normalization of RT-qPCR gene expression data from Actinidia deliciosa leaves infected with Pseudomonas syringae pv. actinidiae
Source: Sci Rep. 2015 Nov 19;5:16961. doi: 10.1038/srep16961 (PMC4652207; doi:10.1038/srep16961)
Supplement: Supplementary Information [file srep16961-s1.doc]

**Reference gene selection for normalization of RTq-PCR gene expression data from *Actinidia deliciosa* leaves infected with *Pseudomonas syringae* pv. *actinidiae***

Milena Petriccione*, Francesco Mastrobuoni, Luigi Zampella and Marco Scortichini

**
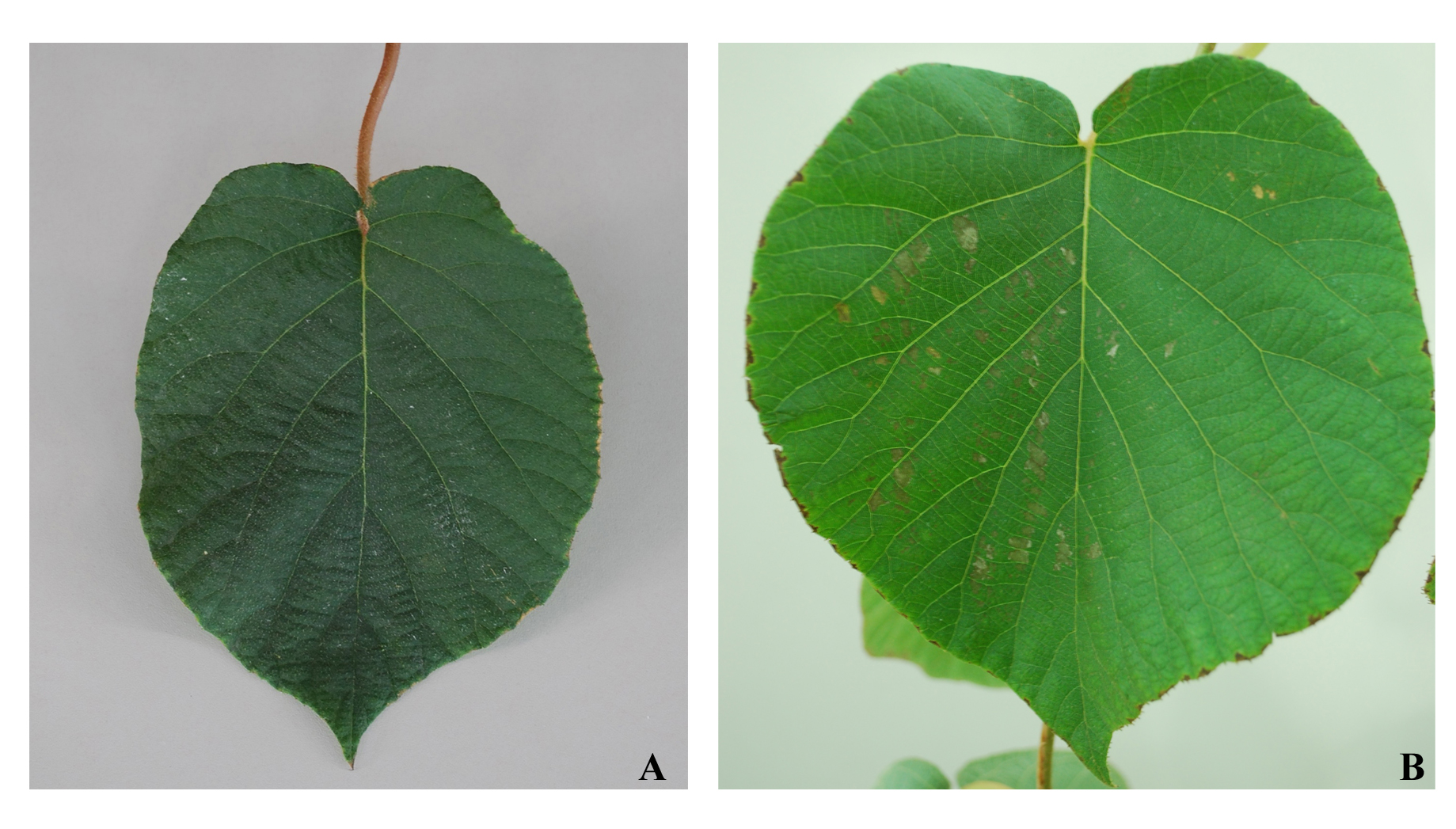
**

**Fig. S1** *Actinidia deliciosa* cv. Hayward leaves nine days after the inoculation with *P. syringae* pv. *actinidiae* CRA-FRU 8.43 at 1-2 x10^3^ cfu/ml (A) and 1-2 x 10^7^ cfu/ml (B).

**Fig. S2** Melting curve for nine candidate reference genes ((*ACT*) *actin*; (*CYP*) *cyclophilin*; (e*EF-1a*) *eukaryotic elongation factor 1α;* (*GAPDH*) *glyceraldehyde-3-phosphate dehydrogenase*; (*GLO7A*) *7s-globulin;* (*PP2A*) *protein phosphatase 2A;* (*SAND*) *SAND family protein*; (*TUB*) *β-tubulin;* (UBC9) *ubiquitin conjugating enzyme 9*) with single peak obtained from three technical replicates of different cDNA pools along with no template control.

**
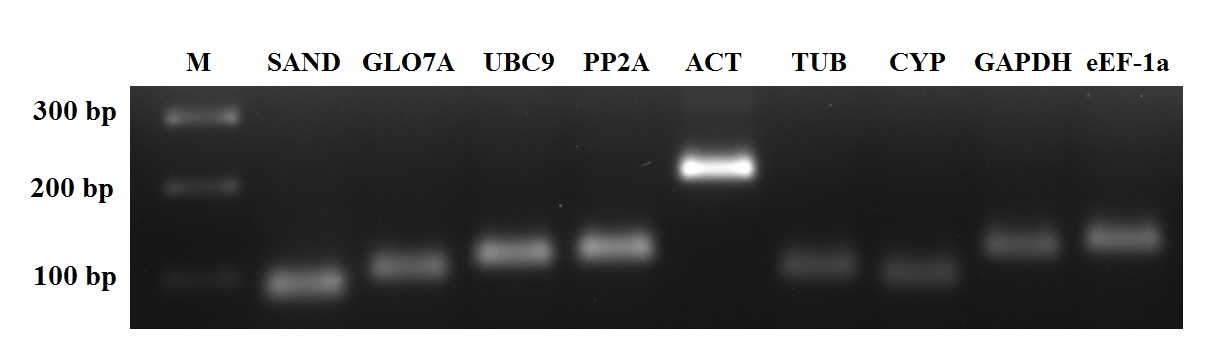
**

**Fig. S3** Agarose gel electrophoresis showing specific RT-PCR products of the expected size for each reference gene obtained by real-time PCR using cDNA as template. (M) DNA marker; (*SAND*) *SAND family protein*; (*GLO7A*) *7s-globulin*; (UBC9) *ubiquitin conjugating enzyme 9*; (*PP2A*) *protein phosphatase 2*; (*ACT*) *actin*; (*TUB*) *β-tubulin*; (*CYP*) *cyclophilin*; (*GAPDH*) *glyceraldehyde-3-phosphate dehydrogenase*; (*eEF-1a*) *eukaryoticelongation factor 1α*.
